# Habits and customs of crab catchers in southern Bahia, Brazil

**DOI:** 10.1186/s13002-017-0174-7

**Published:** 2017-08-23

**Authors:** Angélica M. S. Firmo, Mônica M. P. Tognella, Gabrielle D. Tenório, Raynner R. D. Barboza, Rômulo R. N. Alves

**Affiliations:** 1 Departamento de Ciências Biológicas, Programa de Pós Graduação em Ciências Biológicas (Biologia Animal), Universidade Federal do Espírito Santo, Mucuri, Brazil; 2 Departamento de Ciências Agrárias e Biológicas, Programa de Pós Graduação em Biodiversidade Tropical (Ecologia), Universidade Federal do Espírito Santo/Centro Universitário Norte do Espírito Santo–UFES/CEUNES, São Mateus, Brazil; 3 Departamento de Biologia, Programa de Pós-Graduação em Ciências Biológicas (Zoologia) Universidade Estadual da Paraíba–UEPB, Paraíba, Brazil; 4 Departamento de Ciências Agrárias e Biológicas, Universidade Federal do Espírito Santo, Centro Universitário Norte do Espírito Santo, UFES/CEUNES. São Mateus, Espírito Santo, 29932-540 Brazil

**Keywords:** Trapping, Traditional culture, *Ucides cordatus*, Mangrove forests

## Abstract

**Background:**

Brazilian mangrove forests are widely distributed along the coast and exploited by groups of people with customs and habits as diverse as the biology of the mangrove ecosystems. This study identifies different methods of extracting crabs that inhabit the mangrove belts; some of these activities, such as catching individual crabs by hand, are aimed at maintaining natural stocks of this species in Mucuri (south Bahia), Brazil.

**Methods:**

In the studied community, illegal hunting activities that violate Brazilian legislation limiting the use of tangle-netting in mangrove ecosystem were observed.

**Results:**

According to our observations, fishermen, to catch individual crabs, use the tangle-netting technique seeking to increase income and are from families that have no tradition of extraction.

**Conclusions:**

This analysis leads us to conclude that catchers from economically marginalised social groups enter mangroves for purposes of survival rather than for purposes of subsistence, because the catching by tangle-netting is a predatory technique. Tangle-netting  technique increase caught but also increases their mortality rate. We emphasise that traditional catching methods are unique to Brazil and that manual capturing of crab should be preserved through public policies aimed at maintaining the crab population.

## Background

Mangrove forests have played a historically important role in fishing communities [1, 2] in several regions of the world, and the resources found in these mangrove belts are critical to the survival of these communities [3–6]. Two-thirds of the global fishing population depend on the mangrove ecosystem [7]. Thus, mangrove forests have important ecological, social, economic and cultural significance and represent major sources of income for coastal communities [8].

Several cultures that live along the Brazilian coast depend on coastal resources and have social habits and customs that are linked to the mangrove ecosystem. These cultures are different from the traditional groups known locally as “caiçaras” and “açorianos”, which are located in southeastern and southern parts of the country [9, 10], and have their own ways of using coastal resources for a living. In other regions, the social relationships of cultures intimately associated with mangrove belts have been transformed by the arrival of catchers with no prior experience with extraction procedures. The sea workers that capture mangrove organisms are typically culturally, socially and economically marginalised. Traditional knowledge is poorly incorporated into public policy, leading to a degradation of fishing culture and the entry of people with a weak link to the exploited resource.

*Brachyura* crustaceans, which are the most abundant animals found in the mangrove ecosystem [11], have great economic importance to the communities that live in estuarine areas [12–14]. The mangrove crab (*Ucides cordatus*) (Linnaeus, 1763) is a particularly important economic and subsistence resource found in all Brazilian mangrove forests [5, 15–24]. The catching of this species represents one of the oldest mangrove extraction practices [25, 26] and the most important mangrove-associated economic activity performed on a commercial scale in Brazil [27], generating approximately U.S.$ 9,469.69 per hectare of mangrove [28]. Mangrove crab fishing has cultural, historical and nutritional significance, and the crab is a source of animal protein for human consumption [29].

Popularly known in Brazil as the “*uçá* crab”, “*castanhão*”, “true crab”, “legitimate crab” or “common crab” [30–34], *Ucides cordatus* (Linnaeus, 1763) lives in an area ranging from Florida in the United States to Santa Catarina in southern Brazil [35]. This crab is a semi-terrestrial species that lives mainly in intertidal areas, inhabiting the higher portions of the mangrove region in burrows dug in the muddy substrate at depths of 0.6 to 1.6 m [26, 36, 37]. As the species with the greatest volume of biomass in the mangrove forest [38], *U. cordatus* is of great ecological importance and it is considered an important bioindicator of environmental pollution [39], because of its sensitivity to various pollutants.

Crab catchers, also known as “crabbers” or “shellfish catchers”, are intimately associated with the mangrove environment [40, 41] and sources of ethological, biological and ecological knowledge of the fishery resources with which they interact [22, 42–45]. Because their livelihood depends on mangrove resources, these communities have developed an extensive knowledge of the biotic and abiotic components that make up this ecosystem [21]. In recent years, public policy has used this knowledge to formulate management plans to promote a more sustainable exploitation of mangrove resources [15, 21].

In this context, this study analyses the ethno-ecological aspects related to the capture and sale of the mangrove crab (*U. cordatus*) and evaluates the ethno-ecological knowledge of a community of crab catchers in southern Bahia, Brazil. The aim of this study was to generate information that will contribute to the conservation and management of the species in the region.

## Methods

The names and addresses of crabbers were obtained from the Mucuri Municipal Environmental Department (“Secretaria Municipal de Meio Ambiente de Mucuri”), and the names of catchers registered as professional catchers were obtained from the Mucuri Association of Crab Catchers (“Associação de Catadores de Caranguejos de Mucuri”-ACAM). Eighteen catchers were enrolled in the study; subsequently, 16 additional catchers not registered with ACAM were recruited via the snowball technique. This technique consists in recruiting additional participants who are nominated by other participants according to their levels of experience (culturally competent individuals, that is leaders) [46] until the desired subjects are found. The desired subjects consider themselves experts and are considered experts by their companions [47].

Between the months of June and August 2010, to establish mutual trust between the interviewer and interviewee and thereby preclude the development of a barrier that could undermine the future progress of the study and to understand the organisational structure of this community, relationships were developed with the catchers via free-form interviews. Between the months of September 2010 and January 2011, visits were conducted every 15 days to collect data on the socioeconomic profile of the community, the capture process, the commercialisation of *U. cordatus* and the community’s ethno-ecological knowledge of the species.

Semi-structured interviews on the commercialisation of mangrove crabs were conducted. During these interviews, information on the commercial trajectory of these crabs from their origin in the Mucuri mangrove to their final destination (consumer or business) was obtained. Traders (five owners of establishments) and a former crab catcher who currently resells crabs in other cities and states were also interviewed to collect additional information on this topic.

Information related to the ethno-ecological knowledge of *U. cordatus* was obtained with semi-structured interviews (open-ended questions) with the same 34 catchers who participated in the socioeconomic survey.

In addition to the interviews, six guided tours were conducted with three catchers inside the mangrove employing the technique of Spradley [48]; more specifically, the researcher accompanies the subjects who demonstrate their knowledge of factors of interest in their field of work. At this stage, the techniques used to capture the crabs were observed and the subjects’ knowledge of the biology and ecology of the species was assessed by surveillance and personal inquiries. These catchers were also recruited using the snowball technique.

Because many questions could not be answered by the interviews only, direct observation, also known as participant observation, was also conducted; this technique consists in observing and free-recording information on the activity of interest in the field.

Data were mainly analysed qualitatively; the interview discourses were interpreted [49] using several individual models [50] that consider all of the information provided by all of the informants. To verify the validity of the collected information, two control techniques involving synchronous situations (same question to different people at similar times) and diachronic situations (same question to the same person at very different times) were used according to the recommendation of Marques [51]. These data were also analysed quantitatively; more specifically, simple descriptive statistics and mean values were calculated.

This study was approved by the ethics and research committee of the Federal University of Espírito Santo/Northern Espírito Santo University Center (“Universidade Federal do Espírito Santo/Centro UniversitárioNorte do Espírito Santo”-UFES/CEUNES). The interviews were always performed during previously scheduled visits at the house of the crab catcher or at prearranged locations and preceded by the introduction of the interviewer to the subject and a brief explanation of the purpose of the study. Data provided by the informants were recorded in separate answer sheets, transcribed in full as text and tables and categorised. A photographic record was made whenever possible, and a signed informed consent form and permission for publication of images was provided by all subjects.

## Results and discussion

### Local production practices

The ages of the 34 crab catchers ranged from 21 to 61 years and averaged 36 years. Most informants (25) reported having practiced crab catching for over 20 years, and all of the informants reported that the mangrove forest and crabs were their main source of income. Twelve catchers reported performing other activities to supplement their income: six also caught the blue land crab (*Cardisoma guainhumi*), four fished, and two worked in coal production. Alves and Nishida [22] also reported that some crab catchers in the Mamanguape River, Paraíba state, performed other activities to supplement their income.

Most catchers (27) are male and accompanied by family members (especially offspring) or friends when catching crabs. When accompanied by friends, each catcher catches his or her own batch of crabs; no division of profits occurs, and the accompanying friends merely serve as company. The opposite occurs with family members; the entire batch is often sold to the same buyer and the profits are divided among the family members. In contrast, Nordi et al. [24] observed that the partnership of catchers extended to the sale of the catch in Várzea Nova, Paraíba state, Brazil, regardless of the existence of a family relationship.

Working in pair, the catchers reaches the location of the catch by canoe-type boats (known locally as “bateras”) that range from four to six meters in length. According to the catchers, the boats have a flat bottom that facilitates movement between the mangrove areas. In most cases, the boats belong to the catchers and are used only by family members. The remaining subjects (10) who did not own boats reported that they only work in areas close to the town and in places accessible by land. In the state of Paraíba, canoe-type boats are more common among artisanal fishermen [52], and it is common to see groups of two or three catchers travelling with these vessels to the crab-catching locations [24]. According to Glaser and Diele [23], in northern Brazil, catchers move between sites by canoes that are commonly contracted by two to eight catchers who are relatives or friends. In Mucuri, according to subjects who had been crab catchers for 10 to 15 years, travel to the mangrove forests was previously performed mostly by canoe; however, these vessels no longer exist in the municipality because none of the large trees needed to build these vessels remain and few individuals know how to build these boats. Over the years, the canoes became old, were sold to make rustic furniture and replaced by “bateras”.

Most of the catchers (24) reported spending 5 days per week catching *U. cordatus,* whereas others reported working three to 4 days. The working day starts with the falling tide, as crabs can more easily be caught during the low tide; approximately 5 h per day are spent catching crabs. Fourteen of the catchers reported spending more than 6 h catching. The catchers have no fixed working hours, and their schedule is set by the flow of the tide [53]. In regions of estuaries and mangrove forests, the main abiotic factors that determine the pattern of fishing activities and the organisation of coastal artisanal fisheries are the tides [4, 22, 44, 54].

Because of large numbers of mosquitoes (popularly known in the region as “maruim” (Diptera: Ceratopogonidae)) and horseflies (Diptera: Tabanidae) in the mangrove region, the catchers usually wear long-sleeved shirts and trousers when working and apply diesel oil to exposed body parts to ward off these insects. In other states of northeastern Brazil, as noted by Botelho et al. [55] and Nordi et al. [24] who studied the southern coast of Pernambuco and the estuary of the Paraíba River, respectively, catchers spread a mixture of cooking oil and kerosene over their body to ward off insects. The majority of respondents reported going to the mangrove barefoot or with any footwear that is available. In contrast, Nordi et al. [24] reported that catchers in Paraíba use shoes made from truck tire inner tubes to protect their feet; similarly, Glaser and Diele [23] reported that catchers in the estuary of the Caeté River, Bragança, Pará state manufacture their own footwear.

**Fig. 1:**
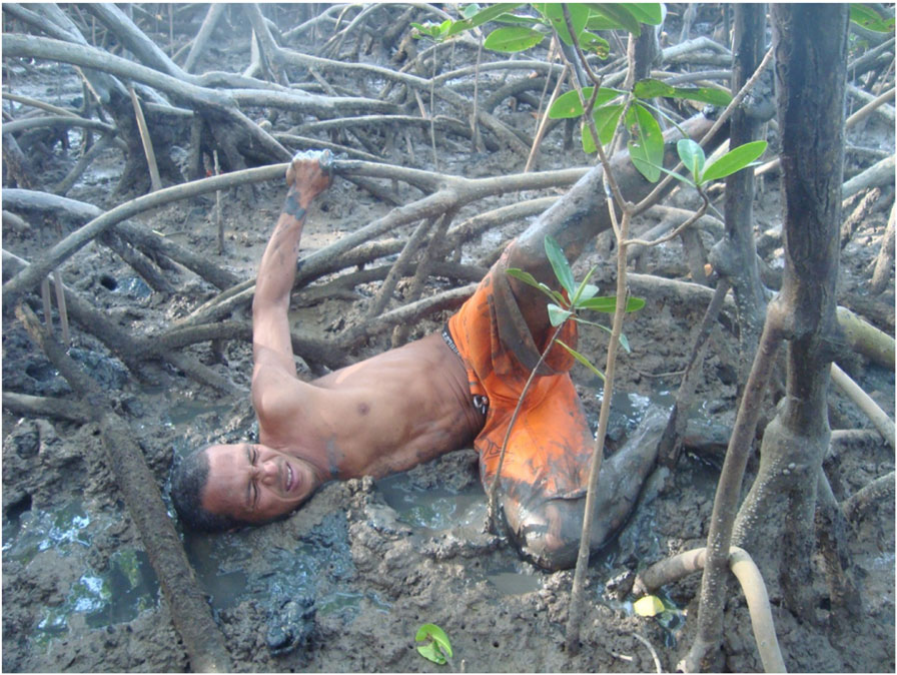
The “braceamento” technique used to capture crabs by traditional catchers

Eighteen of the respondents reported using a “braceamento” technique along with a small net to catch the crabs; 11 of the respondents reported using the “braceamento” technique only and five used a hook technique coupled with the use of a small net (tangle-netting). According to Nordi [15], the “braceamento” technique consists in putting an arm inside the crab burrow, securing the crab by the back part of its shell, pressing its pincers with the thumb and forefinger and pulling it from the burrow in a lateral position (Fig. 1). The “braceamento” technique is the oldest known form of capturing crabs [16] and the most commonly used along the entire Brazilian coast [15] by traditional communities of catchers. Botelho et al. [55] recognised that people who begin crab-catching with no prior experience in mangroves tend to use small nets (small area nets).

**Fig. 2:**
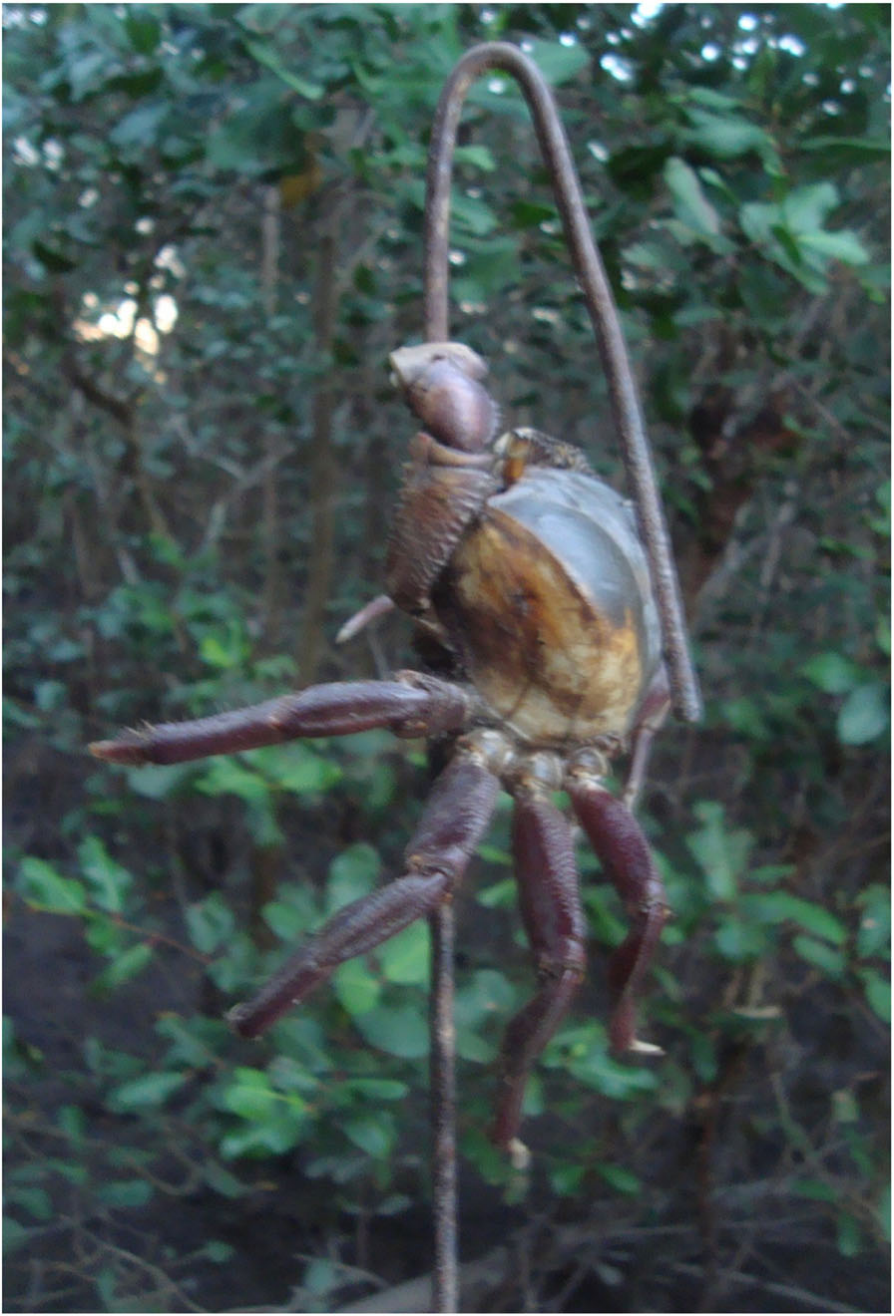
Metal pole (hook) used to pull the crab out by the catchers in Mucuri-Bahia State

The hook technique consists in using a metal pole of approximately 1.5 to 2.0 m in length with a twisted tip that serves to secure the crab. This instrument is inserted into the burrow by the catcher and used to pull the crab out (Fig. 2). The use of this technique reduces the physical effort required for the “braceamento” technique, thereby increasing the productivity of the catcher.

**Fig. 3:**
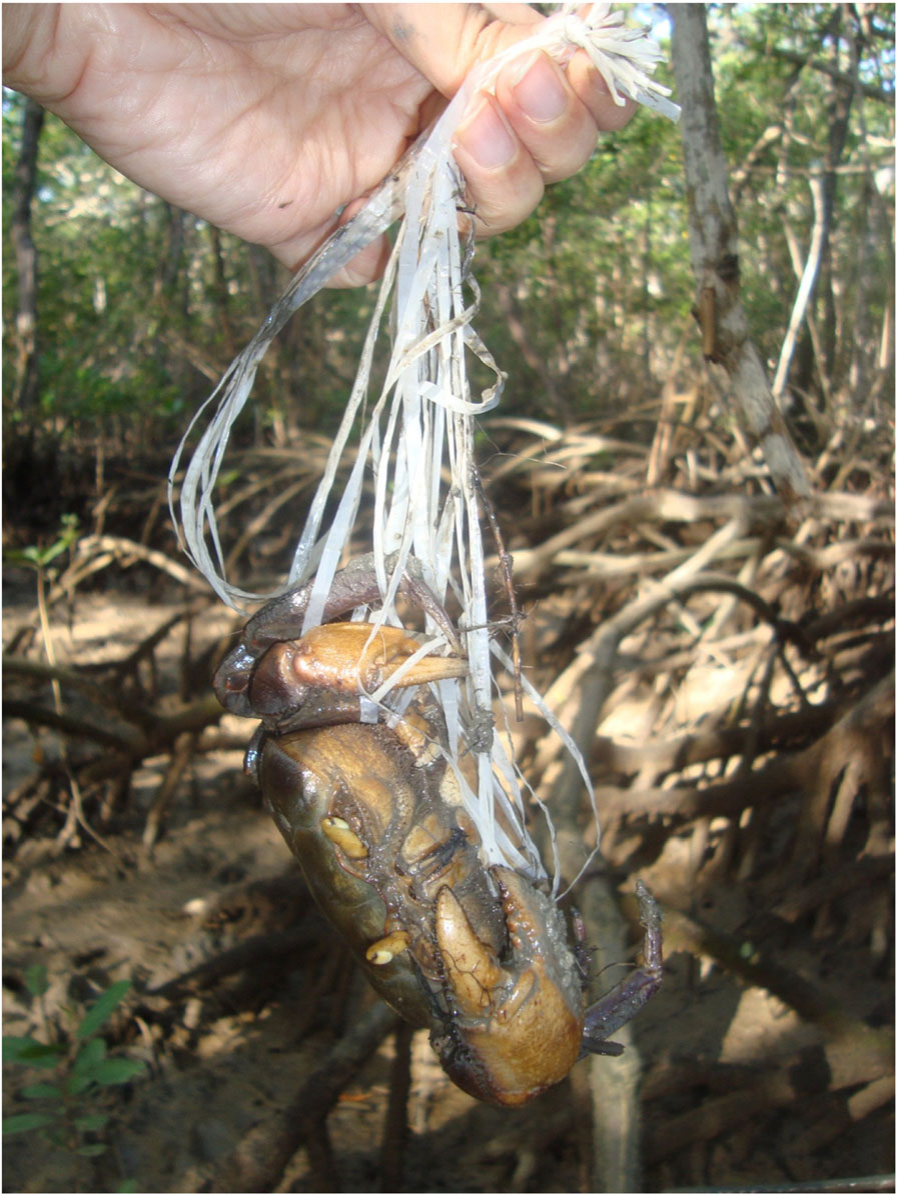
Crab captured by tangle-netting

**Fig. 4:**
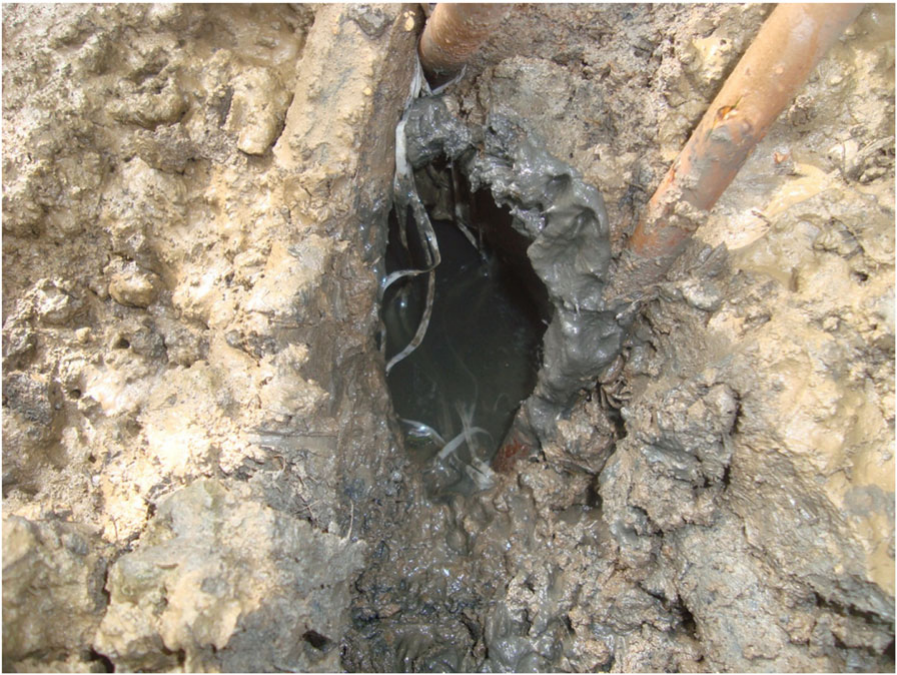
Tangle-netting set at the opening of the burrow to capture the crab

In Mucuri, tangle-netting are traps produced manually using yarn from an artificial raffia bag (made of polypropylene) with two tied ends (Fig. 3). Each end is attached to a piece of *Rhizophora mangle* (L.) root and set at the opening of the burrow to capture the crab (Fig. 4). This technique is similarly performed in several regions [13, 15, 26, 55–57]. As observed by Souto [5] in the fishing community of Acupe, Santo Amaro, Bahia, the choice of technique is, in most cases, related to the season. According to the catchers interviewed in the present study, the crabs begin to moult in the austral winter and thus construct very deep burrows, making it impossible to remove them using arms or hooks. Thus, nearly all informants reported using the tangle-netting during this period.

Because the tangle-netting technique is non-selective (i.e., does not allow for the selection of individuals by sex or minimum capture size) and because many of the traps that are set cannot always be found (and therefore the crabs die), the tangle-netting technique is considered predatory and illegal; moreover, because the material with which the nets are produced does not degrade easily, this technique pollutes the mangrove environment. This technique represents a disruption of traditional capture practices [22], altering the relationship between catchers and the mangrove ecosystem via a reduction in physical contact between the animal and the catcher; moreover, because this technique is easy to learn, the social interaction needed for crabbing is limited [58]; finally, this technique maximises the efficiency of capture, leading to increased pressure on the resources of the mangrove forest.

The catching behaviour of the Mucuri catchers differs during the austral winter, when a tangle-netting is typically used. Instead of remaining within mangrove areas for a long period of time (as the use of both the hook and braceamento techniques require considerable physical effort and time), catchers set approximately 100–150 tangle-netting and retrieve them hours later (or on the following day according to seven of the respondents). This practice is consistent with that described by Alves [56] and Nascimento [58] and raises concerns related to environmental pollution and the excessive mortality of organisms in the mangrove belts.

The Brazilian Institute of Environment and Renewable Natural Resources (Instituto Brasileiro do Meio Ambiente e dos Recursos Naturais Renováveis–IBAMA) ordinance no. 034/03-N of June 2003 allows the capture of mangrove crabs in the northeastern states of Brazil and in the northern regions with “braceamento” and with the aid of a hook or pole only. According to ordinance no. 034, it is a crime to capture crabs using the tangle-netting technique, to capture females and crabs with a carapace less than six centimetres wide in any season and to remove isolated parts such as claws or legs. This ordinance is applied by the inspectors of IBAMA, state environmental military polices and local wardens.

Although this tangle-netting technique is banned, studies by Diele et al. [59] and Passos and Di Beneditto [57] in the Caeté estuary of northern Brazil and in the Gargaú mangrove (Rio de Janeiro), respectively, found that the tangle-netting technique is the primary method of capture used by catchers. According to Nascimento et al. [60], due to its high productivity and ease of use, the tangle-netting technique has spread rapidly throughout most crab catcher communities and threatens traditional capture techniques, the mangrove ecosystem and the *U. cordatus* population.

In the opinion of the tangle-netting users (25), this technique is not predatory or non-selective because the catchers have detailed knowledge of the biology and ecology of the animal that ensures that tangle-netting are placed near the burrows of male specimens of commercial size only; this ability of the catchers to selectively place their nets was confirmed through observations during the guided tours of the mangrove forests. However, it was observed during the guided tours that only a small portion of catchers (14) collected all their tangle-netting at the end of the day. According to the catchers who use this technique, all tangle-netting are collected and none are lost, as the catchers scrape the trunks and roots of *Rhizophora mangle* near the nets with a machete. However, observations of several mangrove forests on the southern coast of Bahia and northern Espirito Santo show that many tangle-netting are not retrieved. Around 5% of the tangle-netting is forgotten in the environment. The use of the tangle-netting technique by catchers that are not part of a family culture of crab catching and who may not have the same knowledge of the ecology of the crab as traditional catchers increases disordered and unmanaged catches.

Throughout most of the year, the catchers capture approximately three to five dozen crabs daily (36–60 crabs); however, during the “walk” season (a term used by local coastal communities that refers to the period during which the crabs leave their burrows to mate), catchers may capture up to 12 dozen crabs (144 crabs); the guided tour and direct observations made during this period confirmed this statement. The catchers control their fishing effort by dozens because it is the way that they sell to the dealer.

According to the catchers, the smaller catches currently caught are due to Lethargic Crab Disease (LCD), a disease that wiped out much of the stock of this species from 2004 to 2009 [61]. This disease is caused by the black yeast *Exophiala cancerae* [62] and has caused widespread mortality of *U. cordatus* in various regions along the Brazilian coast [63, 64]. According to all of the catchers, before the arrival of this disease, up to 100 crabs could be captured daily outside of the mating season. When the disease occurs the catchers cannot sell the crab, its death is quick and there is no sale for dead crabs in the region. The same happens for the capture of females that for whom there is no commercialization.

Soon after being caught, the crabs are placed in polypropylene bags (artificial raffia bags) and taken to the residence of catchers, where, in most cases, they remain until they are sold. Most (24) of the catchers sell all of their catch; only 10 of the catchers reported consuming the crab when no immediate commercial sale occurred. These results were similar to those observed by Fiscarelli and Pinheiro [20] in a study of the socioeconomic profile and ethnobiological knowledge of catchers in Iguape, São Paulo, Brazil.

### Commercialisation

The preferred points of sale for 28 of the catchers are “middlemen” (people who buy production from various localities within a region and then resell in major markets according to Nordi [65] from the state of Espírito Santo and Mucuri (Bahia State). A minority of the catchers sell the crabs from their homes, in their town or at fairs and local markets in nearby towns such as Posto da Mata and Teixeira de Freitas (Bahia). In this case, the crabs are sold in groups of five or six known as “strings” (groupings of five male crabs tied together with polypropylene bag strings or strips of fibres from the stems of a mangrove shrub (*Talipariti tiliaceum* var. *pernambucense* (Arruda) Fryxell) popularly known as “Imbira”). Eight of the catchers reported using “short plastic strings” to tie the crabs together because of their practicality. These short strings are 1.5 m long and typically purchased in a local shop for U.S.$ 3.76 per hundred. In other regions of Brazil such as Iguape, the string is called a “wire” (“fieira”) and consists of a group of 12 crabs; in Fortaleza, Ceará, the string is formed of 10 individuals [66]; in Guanabara Bay, Rio de Janeiro, the string can contain 7–12 animals [67]; and in Paraíba, the string contains 12 individuals [22].

In the municipality of Mucuri, the price for each string varies during the year during the months of December, January and February (i.e., the summer vacation period in Brazil when many tourists frequent the coast (Table 1), thereby increasing trade in the region). Sometimes catchers perform door to door sale. The value of the crab reported in Mucuri was higher than that reported by Magalhães et al. [68] in the municipality of Conde, Bahia, where the price of a string containing five specimens ranges from U.S.$ 1.34 to U.S.$ 1.61. There are many factors involving the price of crabs in the market: quality of mangrove region, crab size and freshness, offer and demand, seasonality.

**Table 1:** Production of crab extraction reported by catchers and small-middlemen

Estimated Production by Interviews		Catcher	Small middlemen	Kiosks	Middlemen	Restaurants
Strings		5 to 6	12	12	12	12
Number of crabs/week		180 to 300	1080 to 1800	72 to 120	3600 to 8400	6000
Price of strings (U.S.$)	Lower	2.68	^a^	1.34	^a^	^a^
	Maximum	3.23	5.37	a	8,06	^a^
	Summer	5.38	^a^	2.15	^a^	^a^
Price of unity of crabs (U.S.$)	Lower	0.47 to 0.53	0.45	1.34	0.67	^a^
	Maximum	0.53 to 0.65	^a^	^a^	^a^	2,68
	Summer	0.90 to 1.1	^a^	2.15	^a^	^a^

^a^ Date not reported

Generally, within Mucuri, crabs are sold by the younger children of the catchers, whereas the catchers themselves sell the crabs in the markets of neighbouring cities. In these cases, most of the buyers are the owners of beach kiosks where food is sold. Due to coastal erosion in Mucuri, only six working beach kiosks were found. Moreover, the kiosks owners reported that these crabs are not a commercial priority for their establishment because of the small profit margin and because the product spoils easily; these kiosks owners tend to prefer more profitable resources, such as fish and marine blue crabs. No sales of isolated parts of the crab or of female specimens (i.e., activities that are prohibited by IBAMA ordinance no. 034) were observed in any of the kiosks. The local wardens and environmental police more frequently act on the commerce as a way to guarantee the effective of the ordinance.

According to all of the catchers, the sale of crabs is becoming unsustainable in the city, as the severe coastal erosion occurring in Mucuri has deterred many of the tourists who are responsible for much of the consumption of this resource. This situation explains why the majority of catchers are no longer selling their production directly to consumers and have no alternative besides to sell crabs to “middlemen”.

This middleman who was interviewed, every delivery is based on trust; no money is given to the catchers at the time of crab delivery, and payments are made only after all of the crabs have been sold. Crabs that die in the transportation are not paid. The transportation takes place at night to avoid the loss of desiccation that can reach in some situation around 45% of the production and to escape from surveillance. According to this middleman, six catchers who are heads of their household (five belonging to the same family) provide approximately 90–150 dozen crabs each week. These crabs are delivered on Fridays to the middleman, packed in cardboard boxes and transported by bus on Saturdays to the town of Vila Velha in the state of Espirito Santo. Crabs are always sold to a large seafood bar in the town. This middleman claimed to be the only former catcher and native of Mucuri acting in this capacity; he reported acting as a middleman because of health problems that made it impossible for him to work as a catcher. He called himself a “small-scale middleman” because of the small quantities he sells and states that his work is of utmost importance because the lack of tourists and shoppers in Mucuri makes him the only purchaser of crabs.

Two other “large-scale middleman” (selling 1000–1500 dozen crabs weekly) were described by the catchers and the “ex-catcher middleman”. One from the city of Vitória, Espírito Santo state sells approximately 300 dozen crabs to the same place (Vila Velha, Espírito Santo) as the “small-scale middleman” and approximately 700 dozen to other commercial seafood establishments in the city of Vitoria. The other “large-scale middleman” is from another municipality of Bahia; however, the catchers were not able to provide additional information on this man. The catchers reported that the two “large-scale middlemen” acquired crabs from neighbouring towns such as Nova Viçosa and Caravelas in addition to the eight families of Mucuri. Selling through a middleman occurs in most cases in southern and southeastern regions because the catchers do not possess the necessary financial means to transport their catch to large consumer centres [29]. Crabs can survive up to a week stored in small areas where they are kept moist.

In the area surveyed, the middlemen are legally required to obtain a control permit from the Municipal Environmental Coordination Office (“Coordenadoria Municipal de Meio Ambiente”) which authorises the transport of crabs within the city and determines the amount that can be transported. This permit is usually sent to IBAMA; however, because there is no IBAMA office in Mucuri, the middleman must take this permit to the nearest IBAMA office in Teixeira de Freitas, Bahia. During data collection for this study, approximately 4000 crabs that were being sold during the breeding season were seized. Even though these crabs were caught outside of the “walk” day, they were being transported and marketed during the “walk day”, which is forbidden by IBAMA ordinance no. 034.

### Income

All of the catchers reported receiving a weekly income of U.S.$ 80.65 to U.S.$ 161.29 from crab catching during most of the year. This income was used for the following purposes, in order of importance: food, household expenses (electricity and water), medicine and clothing. According to Nordi [15], the activity of the crab catcher is characterised as a semi-commercial occupational activity, as it involves the exchange of mangrove crabs for money that is almost immediately used to buy food.

In addition, the catchers reported only collecting large crabs to conserve the stocks of this species and comply with the law as decreed in the third article of IBAMA ordinance no. 34 that prohibits the capture, transport, processing, industrialisation and commercialisation of any *U. cordatus* individual with a carapace less than 6.0 cm wide. All of the catchers stated that they did not collect females at any time of year because of the prohibition against this activity. However, according to the first article of IBAMA ordinance no. 034, the capture, captive maintenance, transport, processing, industrialisation and commercialisation of females of this species is prohibited only from December 1 to May 31. This discrepancy can be explained by the fact that legal information is not passed on to the catchers who continue to adhere to a previous IBAMA ordinance (no. 1208, of 22 November 1989) that prohibited the sale of females. According to Nordi [69], regional studies should be conducted to support the adoption of legal measures against predatory activities and the capture of males and females that are tailored to the unique conditions of each region.

All of the catchers reported that the austral summer is the best season for catching crabs, as the crabs are more numerous and their sale price is higher during this season. Certain catchers capture the mangrove crab during the breeding period, as the lack of enforcement in recent years has made this practice more commonplace not only among members of the community but also among people entering from the city and other municipalities; this practice, which was directly observed by the researchers during the breeding season, puts great pressure on the populations of this species in the Mucuri mangrove. All of the respondents reported being aware of the existence of the breeding period; however, only 21 knew the correct date of the period stipulated in ordinance no. 034. The catchers who did not know the official dates stated that they do not follow the closed season, as it is not applicable to *U. cordatus* in Mucuri. Similar collection practices (i.e., during the breeding season) were reported by Barros [19] in the same study area and by other authors in different regions of the country, including Andrade [43] in Salgado, Pará state, Maneschy [16] along the Amazonian coast, Nordi (44 1994a) in Várzea Nova, Rodrigues et al. (29 2000) in the southeast and south of Brazil, Fiscarelli and Pinheiro [20] in Iguape, Nunes and Samain [70] in Vitória and Souto [71] in the Acupe District, Bahia state.

The “closed” period during which crab-catching is prohibited by the IBAMA through ordinances, laws and normative instructions is when crabs “walk”. In the study area, the closed period is governed by article 2 of IBAMA ordinance no. 034. The prohibition of capture during this period is of extreme importance to preserving the stocks of *U. cordatus* [72]. As was observed by Fiscarelli and Pinheiro [20] in Iguape, more than half of the Mucuri catchers stated that they obtain information related to legislation by speaking with each other, whereas others consult the environmental agencies for clarification. However, according to these catchers, the law is not effective, as the environmental agencies do not adequately enforce the law during this period, and the stipulated date of closure does not match the local breeding season. According to the catchers, the months stipulated in the law are correct; however, the days are not, and breeding occurs days or weeks before the period established by law. The same claim is reported by catchers in Espirito Santo.

In 2011, the dates of the closures were defined according to the largest tidal ranges and phases of the new and full moon in January (from the 5th to the 10th and from the 20th to the 25th, respectively), February (from the 3rd to the 8th and from the 19th to the 24th, respectively) and March (from the 5th to the 10th and from the 20th to the 25th, respectively). This measure is regulated by inter-ministry Normative Act no. 1/2011 of the Ministry of Fisheries and Aquaculture and the Ministry of the Environment and applies to all states where the species lives (Pará, Amapá, Alagoas, Bahia, Ceará, Maranhão, Paraíba, Piauí, Pernambuco, Rio Grande do Norte, Sergipe and Espírito Santo). However, the dates are indicative only and crabs cannot be captured during the stipulated period, even if their breeding season occurs before or after the official dates of closure. Thus, environmental legislation attempts to protect the reproduction of the species no matter when it occurs. During the stipulated period, crabs can only be captured before the start of the closed season and marketed only if they are declared to IBAMA or to the State Department of Environment, depending on the region; the penalty for those who catch or sell the crabs during the closed season is U.S.$ 376.35 to U.S.$ 537.65 plus U.S.$ 10.75 per kilo of crab seized. However, the general public and catchers are generally unaware of or ignore these prohibitive regulations [22, 23, 73].

Most catchers in Mucuri are registered in the Mucuri Association of Crab Catchers (“Associação de Catadores de Caranguejo de Mucuri”-ACAM); however, some are not. According to the catchers, the Association brings no benefits to their lives and their families; in addition, most of these catchers receive benefits from the Mucuri Fishing Colony (“Colônia de Pescadores de Mucuri”) and fear losing these benefits if they are associated with the ACAM. The ACAM was founded in 2009 and currently has 25 full members and 12 members in the registration review process.

### Bioecological knowledge

In Mucuri, *U. cordatus* is popularly known only by the name “crab”. The abandonment of the binomial name (e.g., *uça crab*) in Mucuri was also observed in other regions of Bahia, as reported by Souto [5] in Acupe. According to the catchers, this species occupies “soft” mangroves (mangrove areas under intense tidal influence with a predominance of red mangrove, *Rhizophora mangle*) in burrows (known as dens to the catchers) approximately 1 m deep. Indeed, studies on *U. cordatus* report that the species inhabits areas near the sea with a soft substrate between the average high tide and low tide levels [21] in burrows ranging in depth from 0.5 m to 1.5 m constructed in areas affected by tides [33, 36, 56, 74–76]. According to Hattori [77], mangroves with a predominance of *Rhizophora mangle* showed the highest extraction potential (85.7%) for *U. cordatus*, followed by mangroves with a predominance of *Avicennia schaueriana* (79.3%) and *Laguncularia racemosa* (34.3%). According to the catchers, each den is inhabited by a single crab; however, during the breeding season, it is common to find more than one animal per den. According to Branco [30] and Blankensteyn et al. [78], this behaviour is a function of the territoriality of this species. Catchers distinguish female and male crabs by the form of their abdomen (called “bellybuttom” by catchers). Whereas males have a narrower, more triangular and smaller abdomen, the females (known as “candurua” because their ovigerous form resembles the pouch of the female kangaroo) have a wider, rounded and larger abdomen (known as “apupê”, which refers to where the female crab carries the ovigerous mass). This sexual dimorphism was also reported in other ethno-ecological studies [15, 19, 26, 79] and in other species such as *C. guanhumi* [5, 42, 80–82], *Callinectes* spp. [83] and *Goniopsis cruentata* [84]. Moreover, according to the catchers, males have hairs on their legs that females lack. According to Melo [35], the male mangrove crab has silky hairs, especially on the carpus and propodus. For Nascimento [37], the sex of *U. cordatus* can easily be determined by the external morphology of the species. The respondents also reported that the tracks left by males and females at the entrance of their burrows differ by sex. According to the catchers, the tracks of the males are more visible and deeper because of their claws, whereas the tracks left by the females are more delicate and less visible. Alves et al. [42] also reported that catchers in the Mamanguape river estuary could distinguish the sex of the species by their tracks. According to Pinheiro and Fiscarelli [26], tracks left by males are deeper and “brushed” by the many hairs on the propodus, whereas tracks left by females are much thinner and smoother. According to Mucuri catchers, most crabs have pincers of different sizes; however, “*cuiá*” crabs have equally sized pincers.

All of the catchers reported that the crabs feed mainly on mangrove leaves from red mangroves (*Rhizophora mangle*) that fall in the mud when the tide is low and are carried into the burrows; they also feed on mangrove seeds and roots, corroborating descriptions made by Andrade [43], Leitão and Schwamborn [85], Pinheiro and Fiscarelli [26] and Nunes [70]. Nordhaus and Wolff [86] analysed the stomach contents of *U. cordatus* and found that the diet of this species consists of mangrove leaves (61.2%), unidentified debris and plant material (28.0%), roots (4.9%), sediment (3.3%), bark (2.5%) and animal material (crustaceans, polychaetes, insects, bivalves and gastropods (0.1%)). Branco [30] also analysed the stomach contents of this species and found that the stomachs contained food of plant origin (95%), food of animal origin (53%) and sediment mixed with organic matter (73%); among the foods of plant origin, roots (66%) and bark (51%) were the most frequently encountered items. The difference in stomach content observed between the studies [30, 86] is related to the different methodology used by the respective authors. The first one analysed the data according to the food preference and the second used the frequency distribution of the food item in the stomach. However, the food item is identical for both studies. According to Nascimento [37] and the catchers interviewed in this study, mangrove leaves are the main component of the diet of this species. Asone of the main consumers of mangrove leaf litter [38], *U. cordatus* performs an important role in the ecosystem by cycling nutrients found in the leaves back into the soil, thereby increasing the availability of food for the detritivorous chain [85].

All of the catchers emphasised that there are three important periods in the life of the crab. These periods are directly associated with season and impact the availability of this resource, thereby affecting the catchers’ production practices. According to the catchers, Mucuri has two seasons: the “summer” between October and March and the “winter” between June and September. For the fishing communities of northeastern Brazil, summer is the season with little or no rain and winter is the rainy season [4, 15, 87, 88]. According to the catchers, the reproduction cycle of the crab occurs during the first period and is popularly known in Mucuri as the “walk”. This period is known throughout the Brazilian coast as the crab mating period and is also designated “carnival” or “race” [16, 33, 36, 76, 89–91].

According to the respondents, there are two types of “walks”: the mating walks, when males and females leave their dens to mate, and spawning walks, when females move to the edges of mangroves to wet their “apupê” and release larvae into the water, in puddles or even inside their burrows, especially during ebb tides. Citing Holthuis [92], Ivo and Gesteira [93] suggested that the release of larvae by this species occurs in the sea. However, Nascimento [94] stated that this phenomenon occurs within the dens of females. The so-called “spawning walks” described by the catchers are similar to those reported by Góes et al. [95] who documented the existence of walks specific to ovigerous females that move toward the banks of mangrove rivers and creeks to release their larvae during the ebb tide.

According to all of the informants, the “mating walk” period begins in January and ends in March, whereas the “spawning walk” occurs in late March and/or early April, and both occur during the full or new moon only. Fiscarelli and Pinheiro [20] also reported that catchers from Iguape identified the existence of two types of “walks” and correlated these periods with the same lunar phases. According to the catchers, the first walk in January begins at the first moon (full or new) of the month, lasts approximately 3 days and is repeated every new and full moon (every 15 days) until March. Nunes [70] and Souto [71] also found that walks of *U. cordatus* in Vitória and Acupe, respectively, usually occur during the first full or new moon of January.

According to Diele [96], in the mangroves of the Caeté River, the “walk” lasts one to 3 days and occurs two to 3 days after the full moon. Several studies conducted in northeastern Brazil demonstrated the existence of an extended reproductive period from October to May that varied according to the geographical area in question and always coincided with the warmer times of the year [97]. Nordi [44] reported that populations of crabs in equatorial and subtropical mangroves had different reproductive periods. According to Pinheiro and Ficarelli [26], the “walk” occurs in months with longer photoperiods and higher ambient temperatures and precipitation levels.

The catchers reported that approximately 10 days before “mating walks”, male crabs release white foam that covers the entire body of the animal and serves to attract the female. This foam is actually released at the level of the meropodites by males approximately three to 9 days before mating and is usually spread to the rest of the body with the aid of chelipeds; the foam most likely contains pheromones for sexual attraction [95]. During “mating walks” the capture is prohibited.

The respondents stated that progressively smaller males and females are observed copulating and spawning each year. According to Diele [96] and Pinheiro et al. [98], in the Caeté River Estuary and in Iguape, the female reaches sexual maturity at approximately 2.1 to 3.0 years of age, respectively. After reaching sexual maturity, the crab reproduces once a year [99]. Information on the reproductive period of this species is extremely important for the elaboration of laws establishing periods that are closed to catching aimed at maintaining *U. cordatus* populations [100]. Because “walks” coincide with the summer and a concomitant increase in the flow of tourists (and thus people who consume the crab), effective inspections by the environmental agencies and the establishment of measures prohibiting the capture of this crustacean become even more necessary. Interestingly, 21 of the respondents stated that the population of crabs is currently so small that the crabs do not “walk” as before. According to the catchers, 15 years ago there were so many crabs in the “walk” period that a “wheezing” (a noise caused by the impact of the chelae and legs of the crabs) could be heard emanating from the mangroves, and it was very common to find crabs entering the homes of catchers.

According to the catchers, the second period of the animal’s life cycle is called “fattening”; during this time, the crab is preparing for the third and last period of its life cycle (“shelling” or ecdysis). During fattening, the crabs begin to organise their dens for moulting; the burrows are made deeper, thereby making capture more difficult. During this period, the crab rarely moves around, accumulates fat to be used during moulting [89] and changes colour from blueish to yellowish. In this period, there is little catch due to the quality of the meat and softer carapace, which make the crab more susceptible to death when it is captured.

According to the respondents, the fattening period begins in late May and can run through August depending on when ecdysis begins; because the crabs do not all moult simultaneously, the catchers can continue capturing them during these periods. A similar finding was reported by Nunes [101] in Vitória and by Souto [71] in the Acupe District. According to the catchers, ecdysis begins when the crab closes the entrance to its gallery completely, forming a “mud hill”; this period occurs between August and November, and the largest number of closed dens can be found in September and October.

According to the respondents, the moulting period lasts on average 1 month; Ahmed and Nishida [21] similarly reported that the moulting period of the species is 28 to 29 days. The catchers reported that during the moulting period, the crab is soft and produces a milky white substance; thus, they are called “milk crabs”. This substance consists of chemical compounds (hormones, lipids, proteins, phosphorus, sodium, potassium, calcium, nitrogen, magnesium, copper, zinc, chromium and manganese) that will be used to form the new exoskeleton [37].

Because of the high content of carbonates in the viscera of the crab during this period, the crab is considered unfit for human consumption and may cause side effects such as abdominal pain, changes in the nervous system, lethargy and numbness [26]. According to the respondents, this period was cited as the worst time to capture and sell the crab.

The respondents also noted that the crab changes its shell once a year. In contrast, Diele [91] and Pinheiro et al. [98] reported that this phenomenon may occur more often depending on the age of the individuals, with young crabs moulting more often than larger individuals that moult only once a year. According to the catchers, the mangrove crabs reach a commercially viable size when they are between 3 and 4 years of age; in contrast, other authors have reported different numbers of years, including 10 years [37], 7.5 to 10 years [96], 3.8 years [98] and 11 years [102].

Most of the respondents (25) reported that they could distinguish the sex of the crab by the shape of the plugged area (“hill”) of the dens during moulting. According to them, the “hill” made by males is larger and taller than the “hill” made by females because males remove more mud from their galleries. This phenomenon was confirmed by researchers during the guided tours (during which some of the crabs that were caught were moulting). The indigenous knowledge of moulting is directly linked to the behaviour of crab catchers [71].

The three periods of the life cycle of the animal become clear in one of the catcher’s reports:

*“The crab has three important periods: during fattening, it becomes fat, dark yellow, and a little ugly for 3 months (all winter months without the letter “r”: June, July and August) and it can vary when it starts to lose the shell, as they do not get fat or moult all at the same time, some begin before others. In these days, the crab starts to clean its shell and eats many leaves. The moulting begins when the crabs cover their dens and run from August until November, but more dens are covered in September and October. In these days, the fat turns into milk, and they become soft and milky; if the shell is broken, a bitter milk is released, like the crab blood, only it's white. Each crab stays closed up in the den for a month and then leaves the den with a new shell, skinny, lighter, bluish, and a little bigger. In January, the male crabs produce foam to call the females; the foam is like a perfume, and in the second half of January, they all start to walk to mate and walk every 15 days during the full moon and new moon, until the last tide of March or early April. Then, the mating walk starts and the female opens the apupê and releases the little eggs on the edge of the ebb tide for them to be carried by the tide. The water becomes like a louse egg soup.”*

**Fig. 5:**
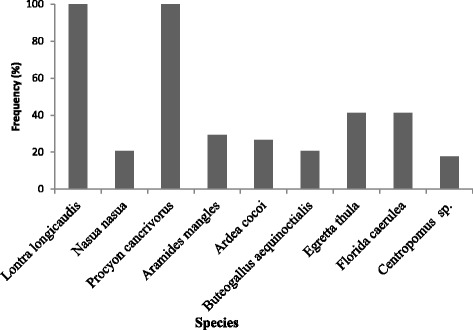
Predators of *Ucides cordatus* reported in the interviews with catchers by frequency of occurrence

The catchers mentioned natural predators of *U. cordatus* (Fig. 5) and during the guided tour some of these predators and/or their tracks were observed in the mangroves. All of the species cited by the Mucuri catchers (Table 2) were also described as predators of crabs in others studies [20, 37, 71]. Insert Fig. 5.

**Table 2:** Production of crab extraction reported by catchers and small-middlemen

Classification	Commun name	Scientific Name	Location	Brazilian State	Source
Mammalia	Fox	*Cerdocyon* *thous*	Mucuri and Aracaju	Bahia and Sergipe	[37]
	Opossum	*Didelphis* *marsupialis*	Mucuri and Aracaju	Bahia and Sergipe	[37]
	Neotropical Otter	*Lontra* *longicaudis*	Mucuri and Iguape	Bahia and São Paulo	This study and [20]
	Coati	*Nasua nasua*	Mucuri and Iguape	Bahia and São Paulo	This study and [20]
	Racoon	*Procyon* *cancrivorus*	Mucuri and Aracaju	Bahia and Sergipe	This study, [37, 71]
Aves	Little Wood Rail	*Aramides* *mangles*	Mucuri and Iguape	Bahia and São Paulo	This study and [20]
	Wood Rail	*Aramides*	Acupe	Bahia	[71]
	Cocoi Heron	*Ardea cocoi*	Mucuri and Iguape	Bahia and São Paulo	This study and [20]
	Mangrove Hawk	*Buteogallus* *aequinoctialis*	Mucuri, Iguape and Aracaju	Bahia, São Paulo and Sergipe	This study, [20, 37]
	Snowy Egret	*Egretta thula*	Mucuri and Iguape	Bahia and São Paulo	This study and [20]
	Light Blue Heron	*Florida caerulea*	Mucuri and Iguape	Bahia and São Paulo	This study and [20]
	Black-crowned Night Heron	*Nycticorax* *nycticorax*	Acupe	Bahia	[71]
	Owl	*Pulsatrix* *perspicillata*	Aracaju	Sergipe	[37]
Pisces	Frillfin Goby	*Bathygobius* *soporator*	Acupe	Bahia	[71]
	Bass	*Centropomus* *sp.*	Mucuri and Iguape	Bahia and São Paulo	This study and [20]
	Atlantic Goliath Grouper	*Epinephelus* *itajara*	Acupe	Bahia	[71]
	Moray Eel	*Gymnothorax* *sp.*	Acupe	Bahia	[71]
	Checkered Puffer	*Sphoeroides* *testudineus*	Acupe	Bahia	[71]
Crustacea	Others crabs		Acupe	Bahia	[71]

According to the Mucuri catchers, mammals feed on adult and young crabs, whereas birds and bass almost always feed on young crabs. Additionally, the catchers reported that the racoon (*Procyon cancrivorus*) is the greatest natural predator of this species because it mimics the catchers when catching the crab, digging in the mud and pulling the crab out of its burrow. According to Souto [5], the catchers of the fishing community of Acupe reported that racoons capture crabs by placing their tails in the burrows and pulling the crabs out when the crabs had grasped their tails. This myth is told in the north of State of Espirito Santo also.

The catchers classified the mangrove soils into two types: 1) the “soft mangrove” (mangrove under intense tidal influence with a predominance of red mangrove, formed mainly of soft mud where the crab lives); and 2) the “dry mangrove” or “salt flats” (sandy mangroves that are not influenced by the tide and are home to the blue land crab *C. guanhumi*). However, 14 catchers also reported the existence of a third type of mangrove known as the “hard mangrove” that is formed of a mixture of mud and sand; this soil is more solid and is home to a crab with a more yellowish colour.

The catchers reported that the crabs consume two varieties of mangroves: the red mangrove (*Rhizophora mangle*) and the white mangrove (*Laguncularia racemosa*). The red mangrove is their major source of food; it has pointed leaves, long seeds, green petioles and its roots and trunks exude a red liquid stain known as “tannins”. The white mangrove has more rounded leaves, red petioles, round seeds and is smaller than the red mangrove. Only eight of the catchers cited another variety of mangrove known as the black mangrove (*Avicennia schaueriana*); this type of mangrove has roots that stick up from the mud in the form of toothpicks, leaves that are whitish on the bottom and a trunk that is smoother than that of other mangroves. The word “mangrove” is generally used to identify different species of tree in the mangrove [103]; however, it can also be used to refer to the set of mangrove trees, i.e., to the vegetation as a whole [5].

When asked to describe natural phenomena that affect the behaviour of *U. cordatus*, all of the catchers cited the lunar phases and the different types of tide. Lunar cycles directly influence the tides, thereby affecting the biological cycles of the species that live in the mangroves and fishing activities in the ecosystem [15, 21, 54, 88]. According to Maneschy [16], all estuarine and coastal zones are influenced by the tidal cycle. According to Nishida et al. [54], catchers from Paraíba use certain terms to designate the tidal variations, including “syzygy tide” or “moon tide” (when the amplitude between low and high tide is highest), “breaking tide” (when the variation in amplitude between the tides begins to decrease), “quarter tide” or “neap tide” (when the amplitude between high and low tide decreases), “dead tide” (when the amplitude between the tides is smallest), “dead water head” (during the last days of dead tides when the moon begins to change, passing to the stage of full or new moon) and “first launching” (when the amplitude of the tides begins to grow, leading to the moon tide). Although the catchers from Mucuri used terms similar as those reported by Nishida et al. [54], only four terms were mentioned by the catchers interviewed in the present study (“launching tide”, “breaking tide”, “dead tide” and “great tide”); the fishermen of the Siribinha community in the state of Bahia who were interviewed by Costa Neto and Marques [104] used similar terms. According to the Mucuri catchers, the “launching tides” occur when the moon goes from crescent to full and from waning to new and last until the “great tide” (syzygy tide) occurs; the “breaking tides” occur when the moon goes from full to waning and from new to crescent until the “dead tides” (“neap tides”) occur.

According to the catchers, the change from the “launching tide” to the “breaking tide” and vice versa occurs every 7 days; the phenomenon then repeats itself when the “great tide” and “neap tide” occur. Because the tide is influenced by the moon 2 days before and 2 days after its apex, the “great tide” lasts 5 days; a similar pattern occurs for the “dead tide”.

The catchers also used the term “tide” to describe the changes in the level of the sea caused by the gravitational pull of the moon. When the tide is at its apex, it is called “high tide”; at its lowest level, it is called “low tide”. According to the catchers, the tides fluctuate over a period of 12 h and 30 min from one tide to another, i.e., from a “low tide” to another “low tide” or from a “high tide” to another “high tide”. The height of the “high tide” and “low tide” (relative to the sea level) also varies depending on the gravitational forces of the moon; when the moon goes from crescent to full and from waning to new, higher tides or “launching tides” (“*it's launching because it releases more*”, Mr. X) occur. The opposite pattern occurs when the moon goes from full to waning and from new to crescent: lower tides or “breaking tides” (“*it's breaking because it breaks more*”, Mr. X) occur during this period. The Mucuri catchers reported that they preferred to capture the crab during the “breaking tide” because the “tide launches” less during this period. A similar situation was described by catchers of Várzea Nova who were interviewed by Nordi [15].

### Recommendations

In 2004, the Brazilian Ministry of the Environment (Ministério do Meio Ambiente-MMA) published the National List of Aquatic Invertebrates and Fish Species that were over-exploited or threatened with over-exploitation through Normative Instruction no. 05/2004 of May 2004. This list contained 11 species of aquatic invertebrates, including three crabs. Thus, in 2011, a national management plan [105] was developed for crustacean species of economic importance (mangrove crab –*Ucides cordatus*, blue land crab –*Cardisoma guanhumi* and Atlantic blue crab –*Callinectes sapidus*). In the case of *U. cordatus*, the plan emphasises the need to maintain the regulation related to the minimum size of crabs that can be captured (6-cm-wide carapaces); in addition, the plan maintains that only the “braceamento” and “tapeamento” techniques with hooks and poles should be used and that the periods coinciding with the “walks” in January, February and March should be designated as closed periods. The plan emphasises that there should be strict control and oversight of these measures. In addition, the plan highlights the need to formulate management strategies for areas of exploitation and the development of studies to define fishery exclusion areas. Finally, the plan emphasises the need to establish packaging standards for the transport of live crabs (which are currently non-existent) and to promote the participation of mangrove crab catchers in the process of shared management of the use of this resource.

The national management plan only protected *Ucides cordatus* as it has an important contribution to the economy. This species, furthermore, is an important source of scientific studies that improve our knowledge of UV radiation impact to human activities [106], environmental organic pollution [27] and metal contamination [107]. Another important point is that the wide distribution of *U. cordatus* along the Brazilian coast, resulting in population of different age and size composition as well as numbers of crabs per mangrove area, physiology, behaviour and reproductive period of crabs [13, 17, 29, 30, 33, 36]. This complicates relation between inspectors and human scavengers and makes interaction between the two difficult.

## Conclusions

That traditional crab caught is a source of results obtained in the field research highlight the fact that the traditional crab-catching are a source of economic subsistence to the local people. Most of the people living on mangrove resources have few employment options in their locality. The catch activity generates 1 week income corresponding to 1/3 of the current minimum wage in Brazil in 2011. The middlemen contribute to local economy and sustainability of the catchers.

An important point to consider is that the catchers appear to have in-depth ethnobioecological knowledge of *U. cordatus*. It is clear that the inclusion of the community in the formulation of strategic decisions on the management and conservation of this species is an important aspect to consider by decision makers.

The variability of “walking mats” and reproduction periods makes it difficult to enforce the regulations along the Brazilian coast.

Our field observations show that the tangle-netting technique causes death of the crab and pollution to mangrove environments. However, the catchers frequently use it even though it is prohibited.
